# Integrated auditory-vocal embodied training for expressive authentic intonation in Chinese zither performance

**DOI:** 10.3389/fnins.2025.1634142

**Published:** 2025-12-18

**Authors:** He Jiang, Hui Lu

**Affiliations:** 1 College of Music and Dance, Guangzhou University, Guangzhou, China; 2 Faculty of Social Sciences and Liberal Arts, UCSI University, Kuala Lumpur, Malaysia

**Keywords:** auditory training, embodiment, pitch perception, traditional music education, zither

## Abstract

**Background:**

Chinese zither music relies on culturally specific microtones (e. g., FA+ and TI–) that differ from Western equal temperament, yet prolonged exposure to standardized tuning often leads to the erosion of these traditional intonation practices. While embodied cognition theory emphasizes the role of sensorimotor integration in learning, its application to the acquisition of non-fixed pitch systems in intangible cultural heritage music remains underexplored.

**Objective:**

This study aimed to examine whether a structured auditory-vocal embodied training protocol—integrating listening, vocal imitation, and metaphoric gestures—could enhance zither learners' accuracy in producing traditional microtones, and to provide empirical evidence for the effectiveness of embodied cognition theory in sustaining culturally authentic musical intonation.

**Methods:**

A quasi-experimental design was employed with 63 intermediate-level zither students. Participants were assigned to an experimental group, which completed a 1-week auditory-vocal-gestural training program, or a control group, which continued regular practice without multimodal integration. Pitch accuracy on two critical tones (FA+ and TI–) was assessed through pre- and post-tests.

**Results:**

The experimental group demonstrated significantly greater improvement in Chinese-style pitch accuracy compared to the control group, along with a marked shift in intonation perception away from equal-tempered biases.

**Conclusion:**

The findings indicate that multisensory embodied training, grounded in the principles of perceptual-motor coupling and gestural simulation, effectively promotes the internalization of traditional microtonal intonation. This study not only offers a novel pedagogical approach for preserving intangible cultural heritage in music education but also substantiates the role of embodied cognition in acquiring culturally situated sensorimotor skills.

## 1 Introduction

A telling phenomenon in the pedagogy of the zither (guzheng) is the frequent, unconscious assimilation of its culturally specific microtones—such as FA+ (slightly raised Fa) and TI– (slightly lowered Si)—into the stable pitches of Western equal temperament during performance. This subtle shift diminishes the authentic expressive color and stylistic integrity of traditional pieces (Chow, 2019; Zhao, 2010). Neuroscientifically, this tendency may be understood as a reflection of long-term auditory cortical plasticity, where pervasive exposure to the standardized tuning of modern education and media reinforces neural representations and sensorimotor mappings for equal-tempered intervals, potentially at the expense of the precise encoding required for traditional intonation (Thrasher, 2008). This creates a core pedagogical conflict between preserving intricate, non-fixed pitch systems and operating within a dominant educational paradigm rooted in Western notation and tuning.

In recent years, the application of embodiment theory in music learning and performance has emerged as a significant research focus. Leman's (2007) systematic approach provides a theoretical framework for this field, suggesting that the body mediates music learning by connecting external sound signals, the body's perceptual system, and internal musical representation and experience. This systematic approach contrasts sharply with traditional music education, which often emphasizes technique and notation, highlighting the body's critical role in musical perception and expression. Through bodily movements, postures, and tactile interactions with instruments, performers can better perceive pitch and rhythm, thereby enhancing musical expressiveness (Jiang and Wu, 2025; Mainsbridge, 2018). This approach deepens the understanding and mastery of music. Although the application of embodiment theory in music performance and education has garnered increasing attention, current research primarily focuses on enhancing musical cognition, perception, and performance skills (Daly, 2022; Van Der Merwe, 2015; Fortuna and Nijs, 2022). However, intonation remains a core challenge in string instrument performance. Unlike other instruments, string instruments require performers to be acutely sensitive to subtle pitch variations and to adjust precisely; string performers must possess a highly developed ability to recognize and regulate pitch (Baugh, 2020). While embodiment theory has been explored to improve performance skills, research on applying this theory to address intonation issues is relatively scarce, particularly in the field of traditional string instruments.

The Zither, as a distinctive traditional string instrument, presents a particularly complex challenge in terms of intonation. Its unique pentatonic scale, which is marked with numbers, significantly differs from the Western music system's twelve-tone equal temperament. The use of non-fixed pitches such as FA^+^ and TI^−^ in traditional zither compositions, which differ from the FA, TI pitches in the twelve-tone equal temperament system, contrasts with the primary tonal system of Western music. In Chinese folk music, fa is usually denoted as FA^+^, and it is usually denoted as TI^−^ to distinguish it from FA and TI in the twelve equal temperaments (Thrasher, 2008). The unique challenges of intonation in traditional Chinese zither music are a testament to the rich and complex nature of this art form. Current teaching and research on intonation primarily focus on the Western music context, neglecting the unique needs of non-Western instruments. Chow (2019) noted that with the widespread adoption of Western music notation following the reform of Chinese folk music in the twentieth century, traditional folk music tuning practices are increasingly aligning with Western tuning standards. This shift not only makes it challenging to express the unique pitch variations of traditional music accurately but also represents an issue prevalent across China yet rarely explored in research (Zhao, 2010).

Instrumental teaching continues to rely predominantly on verbal communication, particularly within traditional master-apprentice teaching models. However, this reliance often overlooks opportunities to cultivate learners' autonomous regulation abilities. Within this teaching framework, students often struggle to effectively adjust intonation during independent practice without the guidance of a teacher. This reveals a deficiency in traditional teaching methods concerning the development of self-regulation skills. It is crucial to recognize the importance of self-regulation skills in music education. Embodiment theory emphasizes the critical role of the body in cognition, suggesting that auditory perception and vocal imitation can serve as vital means for learners to self-regulate. In fact, auditory perception and vocal imitation function as an external feedback system akin to private singing, which aids learners in internal reflection and self-regulation during practice. This regulatory strategy not only helps performers reproduce pitch through vocalization but also internalizes pitch through the interconnection of the body, voice, and emotion.

This study is grounded in the core proposition of Embodied Cognition Theory: cognition is not abstract symbolic manipulation occurring solely in the brain, but is embodied in the body and dependent on real-time interactions between the body and the environment (Varela, 1991; Shapiro, 2011). In music learning, this implies that the acquisition and representation of concepts such as pitch and rhythm are inseparable from the multimodal coupling and coordination of perceptual systems, motor systems (e.g., vocal organs, gestures), and bodily sensations (Leman, 2007; Van Der Schyff et al., 2018). However, most existing research on embodied music cognition has focused on Western music systems or general performance skills, failing to thoroughly explore how embodied training addresses the challenge of precise sensorimotor matching for “non-fixed pitches” in specific cultural contexts. Therefore, this study aims to design a training protocol based on auditory-vocal-gestural multimodal integration to examine the effectiveness of embodied cognition principles in helping learners internalize micro-pitches in traditional Chinese zither music. This is not merely a pedagogical experiment, but also a theoretical inquiry into the constitutive role of the body in culture-specific music cognition. The study proposes the following research questions:

RQ1: Does auditory-vocal fusion embodied training effectively enhance the intonation ability of zither learners when performing traditional pieces?

RQ2: How do variations in learners' gender, age, and length of study impact the effectiveness of auditory-vocal fusion embodied training on improving zither intonation ability?

## 2 Literature review

### 2.1 Teaching strategies for performer's intonation

In current music teaching practice, a number of methods have been adopted to improve the intonation accuracy of performers. Singing techniques such as ‘sight-singing exercises' and ‘vocal rehearsals' are often used as methods of correcting intonation (Reifinger, 2018; Springer et al., 2021). However, the effectiveness of singing in improving pitch accuracy depends on its application. According to Morantz (2016), while vocal warm-ups before playing can enhance pitch recognition to some extent, the improvement is not significant. At this time, their attention may be focused more on the social situation than on the goal of the exercise. And this lack of purposeful practice due to shyness leads to reduced pitch training effects (Morgan, 2022). Meanwhile, other studies investigating pre-tuning vocalization suggest a limited contribution to pitch improvement through brief vocal exercises before playing, such as singing single notes or pitches of a specific mode (Morgan, 2022). This suggests that vocalization, whilst in some cases solving pitch problems in instrumentalists, has not shown consistent results in the observed studies. In addition, it is possible that because the short practice time does not allow the students to fully get into the swing of things, and they often fail to focus on the physical feedback to adjust pitch.

Ear training was used as an essential strategy for intonation improvement. The essence of this training lies in augmenting the musician's pitch recognition abilities and sensitivity to sound details through systematic aural exercises and immediate feedback mechanisms, thereby improving the brain's efficiency in parsing and discriminating auditory signals (Crawford, 2019). Studies, such as those by Tas and Usluer (2022), have demonstrated that regular ear and sight-reading training can significantly improve intonation. On the other hand, in practical teaching, the application of auditory training takes various forms, including the use of recorded accompaniments, tuners, adjacent open strings, and tonic drones (Curry, 2011; Hopkins, 2012; Springer et al., 2021; Nápoles et al., 2019). For instance, drone accompaniments represent a novel approach to improving intonation accuracy. A comprehensive survey by Nápoles et al. (2019) found that most learners perceive it as beneficial in enhancing intonation and concentration. Although a plethora of studies confirm the positive effects of auditory-centered training methods on intonation accuracy, these improvements are not absolute. Research by Springer et al. (2021) and Morgan (2022) indicates that while pitch accuracy improves, the extent of improvement is not pronounced. Further analysis reveals that the effectiveness of these training methods varies in different contexts. For younger or less experienced learners, drones might distract rather than enhance their tonal accuracy (Zabanal, 2019). Therefore, this training requires appropriate adjustments and optimization according to different teaching settings and learner backgrounds.

It is important to note that traditional aural and vocal training is sometimes ineffective in improving learners' intonation, especially if it is not done in a purposeful manner. Some learners are often unable to achieve the desired improvement in pitch through aural and vocal training because they do not have a clear understanding of the objectives of the exercises, or because they do not have a sufficient foundation in music to be able to master these methods. However, for learners with a certain level of foundation, purposeful listening as well as vocal exercises can better guide them to focus on their own intonation regulation, thus improving their intonation perception and control.

### 2.2 The application of embodied methods in instrumental performance education

Embodied methods take various forms in music education, with common examples including somatic learning approaches such as Feldenkrais and Dalcroze Eurhythmics, as well as gestural movements. In traditional instrumental performance education, singing and teacher gestures may serve as the more familiar embodied methods. Casas-Mas et al. (2019) conducted an in-depth analysis of embodied cognition in Flamenco guitar learning, revealing the close relationship between bodily movements, gestures, and music learning. The findings indicate that both traditional mimicry and constructivist internalization methods, such as breath regulation through Private Speech or Singing, can be practical tools for self-guidance and self-regulation, aiding learners in making significant progress in memory, understanding musical notation, and emotional expression. Moreover, the gestures provided by instrumental teachers serve as a visual form of embodied method. Paschalidou's (2024) research further highlights that sensory modalities beyond auditory input, including visual perception, can also assist individuals in processing and experiencing musical information at a deeper level. Simones et al. (2015) explored how piano teachers use gestures to communicate musical knowledge with students of varying skill levels, emphasizing the importance of embodied teaching methods. Teacher gestures not only help students master technical skills but also facilitate emotional expression and musical understanding, allowing students to engage in learning more intuitively and interactively, thereby improving their learning outcomes.

In addition to these traditional embodied methods, Dalcroze Eurhythmics also demonstrates its unique advantages in instrumental performance. Ismail et al. (2023) explored a teaching approach that encourages students of traditional percussion instruments, such as the Kompang, to experience musical rhythm and structure through body movement. The study found that only after internalizing these musical elements through bodily rhythm could students effectively apply them to instrumental performance or singing. After a 12-week Dalcroze Eurhythmics course, students' musical coordination abilities, including synchronization between singing and percussion, significantly improved, along with their attention and musical processing capabilities. Additionally, Daly's (2022) research further emphasized the role of Dalcroze Eurhythmics in stimulating student creativity and autonomy. Through physical movement, students gained a deeper understanding of musical structure and emotion, making their performances more vivid and engaging. At the same time, this embodied training significantly reduced students' performance anxiety, enhancing the stability and fluidity of their playing. This suggests that embodied methods not only promote technical progress but also positively influence emotional expression and creativity. Thus, the significance of embodied methods in instrumental performance education is further validated.

### 2.3 The application of embodied methods in vocal performance education

Embodied methods are prevalent in vocal performance education, especially in promoting students' bodily awareness through somatic behaviors. Paparo (2016) employed the Awareness Through Movement lessons from the Feldenkrais Method to help choir students become aware of their posture, breathing, and vocal production. The results demonstrated that embodied methods effectively enhanced students' control over posture, breathing, resonance, vocal range, and pitch, making their singing more coherent and natural. In Paparo's (2022) follow-up study, although still using the Feldenkrais Method, the focus shifted to exploring the individualized characteristics of personal vocal experiences in academic settings. This study emphasized that embodied lessons, through self-discovery, brought personalized benefits to vocal practice, particularly by reducing tension and unnecessary movements while enhancing bodily support, leading to more natural and fluid performances. These effects are similar to those observed in other embodied teaching methods, such as Dalcroze Eurhythmics, as researched by Daly (2022), where somatic learning helps students release tension and improve performance quality. Furthermore, the advantages of embodied gestures in vocal education are evident, particularly in helping children enhance pitch accuracy, vocal support, and expressiveness. Liao and Davidson (2007) investigated the role of gestures and body movements in improving children's singing techniques, finding that gestures helped children better control pitch, especially in large intervals or sustained notes, where gestures provided essential bodily support for long tones, leading to more stable singing. Overall, embodied means significantly improve the music learning experience for learners in a variety of ways, providing a more intuitive and insightful learning path (Jiang and Wu, 2025).

### 2.4 Theoretical framework: embodied cognition and gesture in music learning

This study is grounded in the paradigm of embodied cognition, which contends that cognitive processes—including learning, memory, and conceptual understanding—are fundamentally constituted by bodily states and sensorimotor interactions with the environment (Shapiro, 2011; Varela, 1991). Moving beyond a view of intonation learning as mere auditory memorization, we conceptualize it as the enactive development of a robust, multisensory representation (Prochnow et al., 2017). Our framework is specifically built upon three interrelated pillars of embodied cognition that inform the design of the intervention.

First, Enactive Cognition posits that knowing is a process of active engagement, where perception and action form an inseparable loop (Thompson, 2017). Learning emerges through goal-directed, perceptually guided activity. In our protocol, the mandated sequence of focused listening followed immediately by vocal imitation instantiates this perception-action cycle, where each auditory input directly seeds a motoric response, reinforcing the sensorimotor linkage through repetition.

Second, Conceptual Metaphor Theory and Simulation hold that abstract concepts are understood via mappings from concrete, embodied experiences (Lakoff and Johnson, 1980). Pitch is a quintessential example, universally structured by the spatial metaphors of “HIGH” and “LOW” (Eitan and Timmers, 2010). Gestures provide a means of simulating these metaphors kinesthetically (Gibbs, 2006). The designed iconic gestures for FA+ (a slight upward-outward movement) and TI– (a slight downward-inward movement) are not arbitrary; they are physical instantiations of the spatial-pitch metaphor, designed to strengthen the mental simulation and internal representation of these microtonal targets (Khatin-Zadeh et al., 2023a).

Third, Perception-Action Coupling refers to the neural and functional link between sensory systems and motor systems, where perceiving an event automatically prepares compatible actions (Schütz-Bosbach and Prinz, 2007). Hearing a target pitch primes the laryngeal system for vocal imitation. By adding a consistent, metaphorically aligned gesture, we introduce a parallel and reinforcing motor dimension. This strengthens the overall perception-action coupling by providing an additional, stable motor trace associated with the auditory target, enhancing the specificity and stability of the sensorimotor mapping.

**Figure 1:**
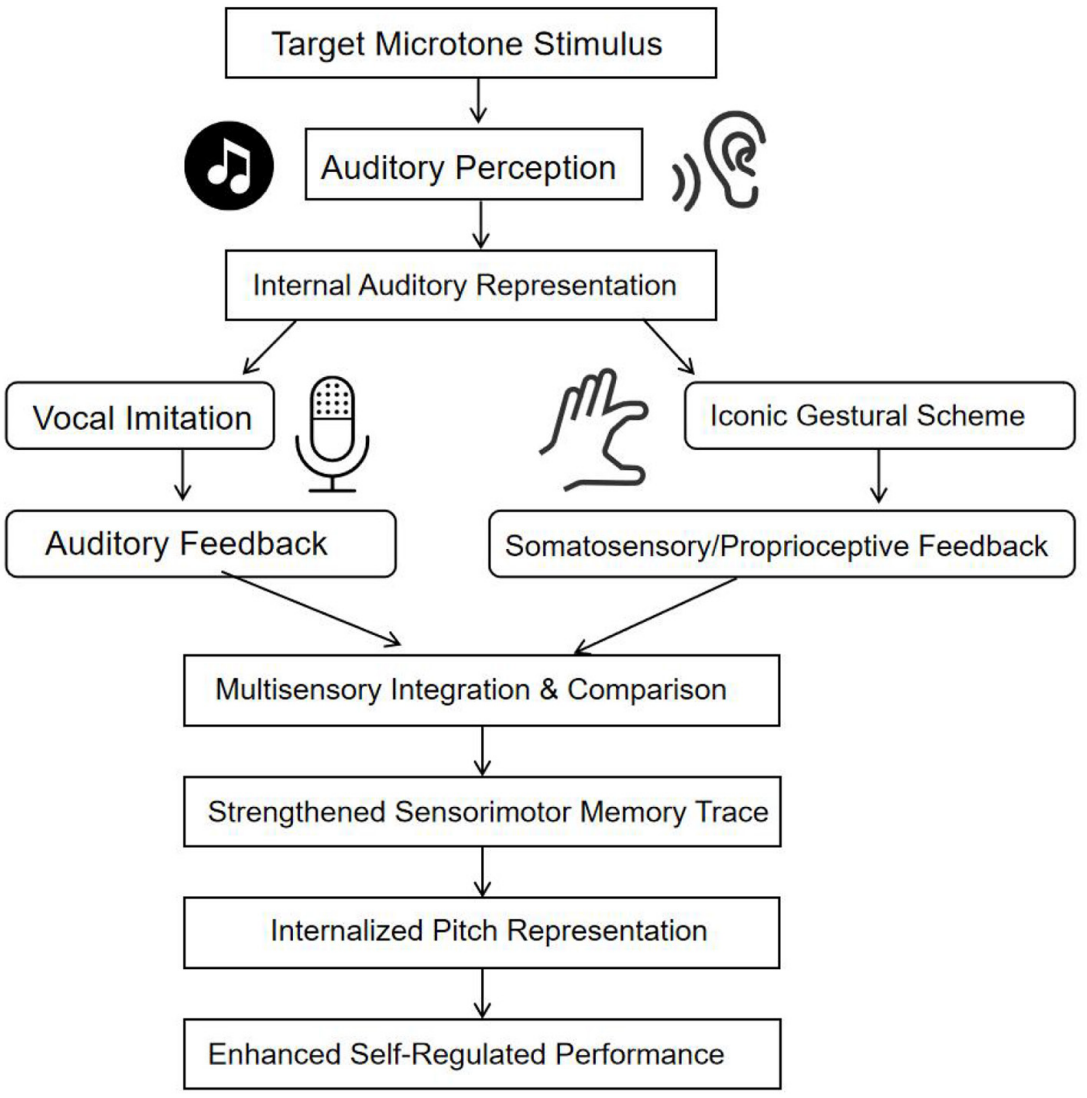
Theoretical model of auditory-vocal integration for pitch internalization.

The integrated Auditory-Vocal Integrated Model (Figure 1) operationalizes these principles. The process begins with Auditory Perception, which, from an enactive perspective, is an active, goal-directed process of attending to and distinguishing the target microtone. This perception activates an internal model that simultaneously guides Vocal Imitation and the execution of a designed Iconic Gestural Scheme. Critically, these are not sequential but synergistic motor simulations, each reinforcing the other. The production of the gesture generates Somatosensory and Proprioceptive Feedback, which is not secondary feedback but a constitutive component of the emerging pitch representation. The continuous integration and comparison of auditory (from voice) and kinesthetic (from gesture) feedback with the internal target facilitates calibration. Through repetition, this Multisensory Integration forges a strong Sensorimotor Memory Trace—a body-anchored “perception-motor schema” that is the embodied cognitive unit of knowledge. Ultimately, this consolidates into an Internalized Pitch Representation that is readily recallable and adjustable, enabling effective Self-Regulated Practice.

Based on this framework, we derive the following testable hypotheses:

H1: Compared to a control group receiving regular technical practice, participants undergoing the multimodal auditory-vocal-gestural training will show significantly greater improvement in the accuracy of identifying and producing the traditional microtones (FA+ and TI–).

H2: The benefits of the training will be evident across diverse learner profiles, as the mechanism of gestural scaffolding taps into fundamental sensorimotor simulation processes.

The theoretical model illustrates the enactive, multisensory process of learning culturally specific pitch. It depicts how active auditory perception initiates a coupled cycle of vocal and gestural simulation, with integrated feedback leading to the development of a consolidated sensorimotor representation that supports independent performance.

In sum, this framework provides the theoretical rationale for hypothesizing that a training protocol explicitly designed to engage multiple, coordinated embodied systems will be more effective than conventional, less integrated methods in developing accurate and internalized representations of traditional intonation.

## 3 Methods

This study used a quasi-experimental design approach, covering both the control and experimental groups, to conduct a before-and-after comparative test.

### 3.1 Research procedures

This study investigates the impact of an intervention on the accuracy of pitch recognition among zither students by comparing an experimental group with a control group. The participants were intermediate-level (Social Art Level Examination Grades 5–7) zither students from educational institutions across three different regions in China. Before the experiment commenced, the research team obtained approval from the ethics committee. It provided detailed information about the study to the participants and their parents, securing written consent from all participants. All participants were added to a specially created social media group for information dissemination and communication. Data transmission complied with the platform's Terms of Service, and all participants were informed of both the method of data transmission and the fact that their data would not be associated with personal identifiers in scientific publications. Prior to the formal commencement of the study, all participants completed a pre-test designed to assess their ability to discern specific pitches involving two pressing techniques in traditional zither repertoire. The pre-test results were used to determine the students' pitch accuracy levels before the intervention.

**Figure 2:**
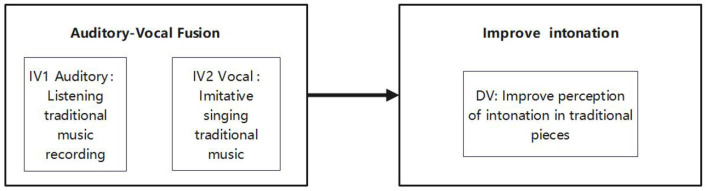
The conceptual framework of the study (IV, Independent variable; DV, Dependent variable).

This study used traditional zither recordings that had been certified by experts as auditory–vocal fusion embodied training materials. Participants in the experimental group were required to engage in imitative singing exercises based on these recordings, thus undergoing auditory-vocal fusion embodied training. This 1-week training was aimed at thoroughly investigating the effect of auditory-vocal fusion embodied training on the learners' perception of intonation in traditional musical pieces (Figure 2).

Following the intervention, the experimental group underwent a week-long training regimen, which included at least six auditory–vocal fusion embodied training daily, with daily check-ins through the social media group. The control group performed the same pieces as the experimental group over the same 7 days, all using the same score. To ensure comparability of practice time and quality, participants in the control group were asked to practice silently, i.e., without any auditory training or sound imitation. Specifically, the control group played the assigned phrase six times per day, focusing on muscle memory and finger technique training without any audio aids or auditory imitation. This design was intended to exclude the influence of listening and singing factors on the effectiveness of the exercises, thus allowing a clearer comparison of the two groups' different performances in pitch perception.

After the experiment concluded, all participants completed a post-test, identical to the pre-test, to assess the impact of the intervention on their pitch recognition abilities. Throughout the experiment, all procedures and communications were recorded to provide a basis for subsequent data analysis and to ensure consistency and accuracy in the experimental process.

### 3.2 Participants

In order to answer the research question, this study was designed to conduct an Experiment with a control group and an experimental group using a pre-test and post-test method. Prior to the beginning of the study, the Research Ethics Committee of a teacher training university in China granted ethical approval. All participants had voluntarily joined the study, and upon obtaining written consent from all of them, the research team added these participants to a specially created social media group. This group served primarily as a platform for information dissemination and communication. In accordance with purposive sampling criteria, thirty-three intermediate zither students were selected for each group. To ensure the validity and generalizability of the data while minimizing potential sampling errors, measures were implemented to guarantee the reliability of the samples. Specifically, the research team collaborated with three educational institutions located in diverse geographic regions to recruit suitable participants. Through oral promotion utilizing the networks of zither teachers at these education centers, the study aimed to attract zither students who had already achieved an intermediate level of performance. Participants were required to be recognized by their zither teachers as having intermediate playing skills, committing to following the training plan within a week and having no hearing or speech impairments.

In the initial phase, we collaborated with zither teachers from these institutions to select a purposive sample of 66 intermediate-level zither learners who had no hearing or speech impairments, with 33 students from each region. These participants were randomly assigned to either the experimental or control group. After random assignment, independent samples *t*-test confirmed no significant differences between the two groups in baseline data such as age and duration of music learning, ensuring intergroup comparability. However, due to mid-study attrition, the final control group comprised 31 participants, including six males and 25 females, while the experimental group consisted of 32 participants, including five males and 27 females. The experimental group had a mean age of 12.562 years, with a standard deviation of 6.88. The control group had a mean age of 13.645 years, with a standard deviation of 6.66.

### 3.3 Intervention design

The intervention in this experiment was designed based on the theory of embodied cognition, emphasizing the enhancement of students' pitch discrimination abilities through the integration of auditory perception and vocalization. Guided by this theory, the students in the experimental group underwent a week-long auditory–vocal fusion embodied training regimen, which included listening and imitation exercises aimed at strengthening their pitch perception and control through multisensory interaction.

**Figure 3:**
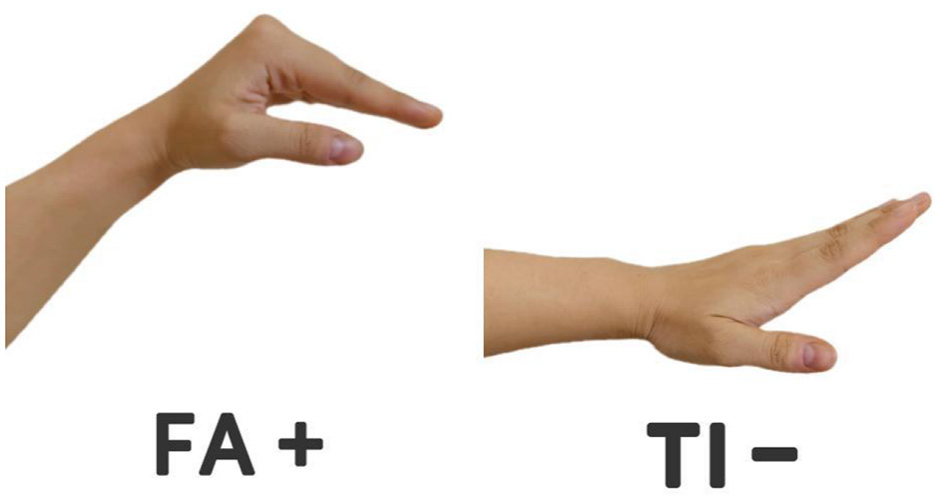
Gestural movements accompanying the singing.

When musical stimuli reach the auditory nerves, the sensorimotor circuits in the brain are activated (Phillips-Silver et al., 2020). The act of listening and singing can be viewed as a process that integrates perception and movement. The ability to accurately perceive pitch is essential for correctly identifying pitches, and this study used traditional zither recordings as auditory–vocal fusion embodied training materials for participants to perform. Subsequently, participants were required to perform imitative singing exercises based on these recordings to reproduce the mechanism of pitch through modeling. When singing involving non-fixed pitch FA+ as well as TI– tones, participants were allowed to use body kinaesthesia to enhance the perception of the modeling. This 1-week training was designed to investigate in depth the effects of auditory and vocal embodied behavioral training on learners' perception of pitch in traditional musical works. As the students sing FA^+^ and TI^−^, make the movements shown in Figure 3 to assist with the singing. Singing is not merely an act of imitating sounds; it also involves the coordination of multiple sensory feedbacks within the body. These bodily feedbacks are transmitted to the brain via the nervous system, aiding students in better calibrating and controlling pitch.

After listening to the audio, participants were required to perform the singing imitation at least six times immediately. During this process, students were encouraged to mimic the pitch they heard precisely and, through repeated practice, gradually increase their sensitivity to pitch accuracy. This process of perceiving music further enhances understanding and perception, thereby creating a feedback loop of interaction with the music (Leman and Maes, 2014). Through this combined practice of listening and singing, participants from the experimental group were able to gradually improve their mastery of pitch accuracy by interacting with their bodies and the environment.

The study considered the control of variables. The first was the students' basic ability. All participants were screened as intermediate-level zither students to ensure that the student's abilities were similar before the intervention and to avoid bias due to differences in skill levels. The second was the practice materials and standards. The experimental group, as well as the control group, were provided with the same learning sheet music. However, the experimental group performed additional listening to the standard audio and was asked to perform no fewer than six modeling exercises on the audio. In contrast, the control group did not listen or sing and maintained a normal daily practice routine.

### 3.4 Detailed description of the auditory-vocal embodied training protocol

The training protocol was designed in strict adherence to the principles of multimodal integration and perception-action coupling in embodied cognition. Learners are required to perform vocal imitation immediately after listening, aiming to establish a tight auditory-vocal motor loop (Phillips-Silver et al., 2020). The specially designed gestures (see Figure 3) are not arbitrary bodily movements but rather grounded in spatial metaphors of pitch (i.e., F+ corresponds to a slight upward lift and abduction of the hands, while B– corresponds to a slight downward descent and adduction of the hands). Their purpose is to provide a stable, repeatable body-anchored reference for the abstract concept of micro-pitches. By repeatedly executing vocalization and gestures in coordination, we anticipate that learners will form enhanced, distributed sensorimotor representations in the brain, thereby enabling more precise internalization and retrieval of target pitches (Khatin-Zadeh et al., 2025). This directly addresses the dilemma in traditional master-apprentice systems where learners lack self-regulatory “bodily tools

Auditory-Vocal Embodied Training: Total training duration (1 week), daily training frequency (at least 6 sessions), and recommended duration per session (10–15 min, including listening and singing). These “6 sessions” should be completed in concentrated time periods. Participants are required to submit a short voice or text feedback for check-in daily, and the research team will verify and document the check-in status to ensure the effective implementation of the intervention.

Specific activities included in each session:

Step 1 (Listening): Participants listen to expert-verified standard pitch recordings (F+ and B–). Details regarding the source of the recordings, performer, recording quality, and playback device/platform must be provided.

Step 2 (Singing and Movement): Participants immediately imitate and sing the heard pitches. The integration of “somatosensory-motor engagement” must be elaborated as follows: When singing F+ and B–, participants are required to perform the specific hand gestures illustrated in Figure 2. The design of these gestures is based on the well-known Curwen Hand Signs in music education, with distinct hand movements that differ from the original Curwen Hand Signs.

Step 3 (Repetition and Internalization): Repeat Steps 1 and 2 at least 6 times. Emphasis is placed on purposeful practice—participants' goal is not mechanical repetition, but rather active comparison, adjustment, and internalization of the target pitches through the linkage of auditory, vocal, and motor modalities.

Overall, this training protocol was designed by the research team based on Embodied Cognition Theory and traditional pedagogical approaches. It is an independently completed home exercise without real-time guidance from teachers or parents.

### 3.5 Measurement

We detailed the arrangements for the pre-test within the group, which included a series of questions distributed via an online platform. These tests were specifically designed to assess the zither students' ability to discern the intonation of two specific pitches that require pressing techniques in traditional music (As there are only two pitches in the zither that require pressing, the test focused on the intonation of these two pitches).

The intonation test consists of six items extracted from recorded segments of traditional pieces. Items 1–2 test the intonation of pitch FA^+^, items 3–4 focus on pitch TI^−^, and items 5–6 provide a comprehensive assessment of intonation recognition (Table 1). Each item offers four options: standard answer, equal temperament, significant deviation, and all the same, to evaluate learners' mastery of traditional piece intonation. After collecting the pre-test data, participants in the experimental group underwent a week-long auditory–vocal fusion embodied training, practicing auditory–vocal fusion embodied training at least six times daily with mandatory daily check-ins in the group for seven consecutive days. Post-training, participants' intonation changes were assessed using the same six test items in an online post-test. Participants could opt out of the experiment at any time, while the control group maintained their regular daily training. After the training period, researchers conducted a post-test online, using the same six intonation test items to assess participants' intonation changes. All operations and communications throughout the study were recorded, providing a reliable basis for subsequent data analysis and ensuring consistency and error control in the experimental process.

**Table 1:** Intonation test item and training details.

**Test items**	**Test content**	**Intonation options**	**Training details**
Item 1–2	Intonation FA^+^	A. Standard Pitch B. Equal Temperament Pitch C. Significantly Deviated Pitch D. All three options sound the same	Practice auditory–vocal fusion embodied training at least six times daily; check-in is required to ensure participation.
Item 3–4	Intonation TI^−^	A. Standard Pitch B. Equal Temperament Pitch C. Significantly Deviated Pitch D. All three options sound the same	Practice auditory–vocal fusion embodied training at least six times daily; check-in is required to ensure participation.
Item 5–6	Comprehensive intonation	A. Standard Pitch B. Equal Temperament Pitch C. Significantly Deviated Pitch D. All three options sound the same	Practice auditory–vocal fusion embodied training at least six times daily; check-in is required to ensure participation.

To ensure the reliability and validity of the test items, this study specially invited domain experts to rate and validate the zither intonation test items. During the validation process, experts generally awarded a score of 4.83 out of 5 for the test content, clarity, and appropriateness of the questionnaire answer options. Furthermore, the audio quality received a score of 4, while the exam items designed to assess participants' ability to discern intonation effectively received a score of 4.63. Therefore, it is demonstrated that the test items comprehensively cover the traditional range of intonation and that the option settings are reasonable. Additionally, the audio clips used for participant training were of high quality and conformed to traditional intonation standards.

### 3.6 Data analysis

In this study, the effectiveness of the intonation auditory-vocal fusion embodied training for zither learners was evaluated primarily by comparing the accuracy rates between the pre-test and post-test. Initially, all pre-test and post-test responses from the participants were collected. It was ensured that all data were complete and accurate, and responses from participants who did not regularly complete the training tasks were excluded. The answers were converted into quantifiable data and entered into the data analysis software IBM SPSS Statistics 27.0.1.

For the RQ1, a normality test was conducted on the total scores of the pre-test. Since the distribution was not normal, the Wilcoxon signed-rank test was employed to examine whether there was a significant difference between the pre-test and post-test scores. The significance level was set at 0.05, and the test results were used to address the research question. Additionally, the McNemar test was used to analyze each item in detail and investigate the impact of auditory–vocal fusion embodied training on the intonation of zither learners. Additionally, addressing the RQ2, the Mann-Whitney U test, and Wilcoxon W test were used to examine the impact of gender on the improvement of intonation. Subsequently, the Kruskal–Wallis H-test was employed to explore whether factors such as age and the length of learning the instrument influenced the enhancement of intonation (Morgan et al., 2019).

## 4 Findings

### 4.1 Descriptive analyses of the results

In the pre-test data analysis, a significant trend emerged, indicating learners' general preference for equal temperament as the correct intonation.

In the study of zither learners' intonation perception tendencies, the first and second items, focusing on pitch FA^+^, showed that most participants in the experimental group chose the equal temperament over the correct option (see Table 2). This pattern was also evident in the control group. Although the number of correct selections slightly increased in the third and fourth items on pitch TI^−^, the preference for equal temperament still dominated. This tendency persisted in the final two overall pitch tests. Across all 63 participants, there was an overall inclination toward equal temperament, particularly in the pitch FA^+^ test, despite some increase in correct selections. This suggests that in zither education, influenced by Western musical education, the equal temperament becomes deeply ingrained, indicating a need for a stronger emphasis on traditional pitch education.

**Table 2:** Option frequency of the experiment group pre-test.

**Option**	**Group**	**Test item**					
		**1**	**2**	**3**	**4**	**5**	**6**
Correct option	Control	7	2	16	6	6	15
	Experimental	3	0	15	8	9	11
Equal temperament option	Control	19	23	13	17	21	16
	Experimental	20	25	12	11	18	13
Other incorrect options	Control	5	6	2	8	4	0
	Experimental	9	7	5	13	5	8
Total options	Control	31	31	31	31	31	31
	Experimental	32	32	32	32	32	32

Therefore, based on the conditions of these two groups, it can be inferred that Zither learners commonly tend to adopt equal temperament as the correct pitch for traditional pieces during their learning process.

### 4.2 Results of Research Question 1

To answer RQ1, we employed the non-parametric Wilcoxon Signed Ranks Test to examine the changes in pre-test and post-test scores between the experimental and control groups. The results indicated that after a week of combined auditory and vocal training, there was a significant improvement in the zither learners' intonation perception of traditional music. Specifically, the difference in scores before and after the test in the experimental group was significant (*Z* = −4.910, *p* < 0.001), suggesting that the participants' intonation tendencies had significantly shifted closer to the zither's standard intonation after the training. In contrast, the difference in scores before and after the test in the control group was not significant (*Z* = –0.229, *p* < 0.819), implying no improvement in intonation. Therefore, these results answered RQ1, indicating that combining auditory and vocal training significantly enhances the intonation accuracy of traditional pieces.

Furthermore, to gain further insight into the participants' performance changes across each item, the McNemar test was applied to statistically analyze the data for each item before and after the training in the experimental group. The results clearly showed a significant improvement in learners' scores on various intonation test items following the training (*p* < 0.05). At an overall level, participants experienced a significant increase in their average scores across all six test items after receiving training, especially notable in the second item regarding the intonation of pitch FA^+^ (see Table 3).

**Table 3:** Pre-test and post-test data for each item in the experimental group (*N* = 32).

**Item**	**Pre-test / Post-test**	**Mean**	***SD***	***f***	**Sig**.	***r***
1	Pre-test	0.47	1.48	3	0.001	0.90
	Post-test	4.53	1.48	29		
2	Pre-test	0	0	0	0.001	0.808
	Post-test	3.28	2.41	21		
3	Pre-test	1.84	2.34	11	0.001	0.732
	Post-test	4.38	1.68	28		
4	Pre-test	1.25	2.2	8	0.001	0.773
	Post-test	4.22	1.84	27		
5	Pre-test	1.41	2.28	9	0.001	0.732
	Post-test	4.06	1.98	26		
6	Pre-test	1.72	2.41	11	0.001	0.79
	Post-test	4.84	0.88	31		

SD, Standard deviation, f, Number of participants with correct answer, r, effect size.

The number of participants who correctly answered this test item increased from zero before training to 21 after training. Considering these outcomes, the intonation test item for pitch FA+ emerged as the most challenging aspect for zither learners, indicating that such intonation skills may require more attention and training in future educational practices.

### 4.3 Results of Research Question 2

In exploring the RQ2, which examined the relationship between improvements in intonation and participants' gender, age, and length of learning time, the zither, an initial statistical analysis was conducted on gender differences. Using the Mann-Whitney U test and Wilcoxon W test, a *Z*-value of −1.012 and a corresponding *p*-value of 0.312 were found, indicating that gender did not have a statistically significant impact on intonation improvement (*p*-value > 0.05). Subsequently, regarding age, participants were divided into three groups: primary school, secondary school, and post-secondary school. The Kruskal–Wallis H-test results showed that although the post-secondary school age group performed better in auditory tests, the differences in intonation improvement among these age groups did not reach statistical significance (*H*-value of 4.534, *p* = 0.104). This suggests that while age is a factor to consider in both education and tests, it is not a dominant factor influencing test performance. Participants were categorized into four groups based on the length of time spent learning the zither: 1–4 years. The results indicated that the length of time spent learning the zither did not have a statistically significant effect on intonation improvement either (*H*-value of 3.024, *p* = 0.388). In conclusion, variations in factors such as gender, age, and length of study do not significantly affect the efficacy of auditory and vocal fusion training in improving intonation.

## 5 Discussion and conclusion

### 5.1 Theoretical implications: embodiment in acquiring cultural-specific pitch

This study demonstrates that a structured auditory-vocal-gestural training protocol significantly enhances zither learners' intonation accuracy for culturally specific microtones. Beyond its pedagogical efficacy, these findings offer substantial insights into embodied cognition theory, particularly regarding how complex, culturally situated sensorimotor skills are acquired and stabilized.

First, the results provide robust empirical support for a core tenet of embodied learning: multimodal, body-grounded practice is superior to unimodal or disembodied approaches. The experimental group's significant improvement over the control group, which relied on conventional technical practice, underscores that learning to perceive and produce non-fixed pitches is not merely an auditory calibration task. Instead, it is facilitated by creating a distributed sensorimotor representation that integrates auditory targets, vocal motor execution, and metaphoric gestures (Leman, 2007). This multisensory integration transforms an abstract perceptual challenge into a concrete, body-anchored activity, leading to more robust and internalized knowledge.

Second, the critical role of gesture as a cognitive scaffold is highlighted. The designed iconic gestures for FA+ and TI– served not as mere aids but as constitutive elements of the learning process. By providing a stable spatial-motor analog for abstract pitch relations, the gestures likely reduced cognitive load and strengthened the mental simulation of the target microtones (Goldin-Meadow and Beilock, 2010; Prochnow et al., 2017). The finding that training effectiveness showed no significant variation across gender, age, or duration of study further suggests that this gestural scaffolding mechanism taps into fundamental, widely shared perceptual-motor simulation processes, operating as a universal cognitive tool for stabilizing intangible concepts.

Our findings can be coherently interpreted through the “4E” cognition framework (Kiverstein and Clark, 2009): (1) Embodied: Accurate pitch knowledge was not stored abstractly but existed in the coordinated patterns of listening, singing, and specific hand movements. (2) Embedded: Learning was successful precisely because the training was embedded within the authentic context of traditional zither microtones (FA+, TI–), countering the dominant embedded context of equal temperament. (3) Extended: During training, the expert audio recordings and the learners' own gestures functioned as extensions of their cognitive system, offloading and supporting the internalization process. (4) Enactive: The improved intonation was not passively absorbed but enacted through the learner's active, repeated cycles of perception (listening) and action (vocalizing/gesturing), continuously refining the sensorimotor loop.

A key, theory-relevant finding is the pronounced pre-test bias toward equal temperament. This default preference exemplifies how a dominant, culturally embedded tuning system can overwrite the sensorimotor mappings for a traditional system (Thrasher, 2008). Our intervention successfully disrupted this default by building new, competing embodied representations, demonstrating the theory's power to explain and remedy the erosion of intangible cultural heritage.

### 5.2 Pedagogical and research implications

The success of this method builds upon the foundation of aural-oral training (Smith, 1995) but crucially advances it by integrating a deliberate gestural component attuned to cultural specificity. This approach moves beyond conventional sight-singing and offers a practical model for “distributed embodiment” in music education (Khatin-Zadeh et al., 2023b), where complex concepts are stabilized across multiple modalities.

The study underscores a critical challenge in music education: the marginalization of non-Western tuning nuances due to pervasive pedagogical frameworks. Future curricula must emphasize such authentic intonation, and teacher training should incorporate embodied methodologies to broaden instructional repertoires. Furthermore, while our sample showed consistent improvement, the applicability of self-regulated embodied training across younger ages or earlier learning stages warrants investigation due to differing self-regulatory capacities (Yaban and Gaschler, 2025).

### 5.3 Limitations and future directions

This study is limited by its focus on intermediate learners and online delivery. Future research should validate these findings with beginners and advanced students in offline settings. To deepen the theoretical contribution, subsequent studies should: (1) Measure the spontaneous use of gestures after training as a behavioral indicator of complete internalization. (2) Employ neuroscientific methods to directly observe the enhanced coupling between auditory and sensorimotor cortices following multimodal embodied training. (3) Explore the development of embodiment schemes for other challenging elements across diverse musical traditions.

### 5.4 Conclusion

This study advances our understanding by providing empirical evidence that embodied cognition theory offers a powerful framework for preserving intangible musical heritage. The auditory-vocal-gestural training protocol not only bridged a pedagogical gap in zither education but also exemplified how cognition, rooted in bodily experience, can be harnessed to reclaim and sustain culturally specific perceptual-motor skills. This affirms the vital role of the body in musical knowing and offers a promising pathway for the future of culturally responsive music education.

## Data Availability

The datasets presented in this study can be found in online repositories. The names of the repository/repositories and accession number(s) can be found below: The data presented in this manuscript has been made openly available. Test audio materials available at https://doi.org/10.6084/m9.figshare.27075490.v1. Test results available at https://doi.org/10.6084/m9.figshare.27075484.v1.
